# Accuracy of Five Serologic Tests for the Follow up of *Strongyloides stercoralis* Infection

**DOI:** 10.1371/journal.pntd.0003491

**Published:** 2015-02-10

**Authors:** Dora Buonfrate, Marco Sequi, Rojelio Mejia, Ruben O. Cimino, Alejandro J. Krolewiecki, Marco Albonico, Monica Degani, Stefano Tais, Andrea Angheben, Ana Requena-Mendez, José Muñoz, Thomas B. Nutman, Zeno Bisoffi

**Affiliations:** 1 Center for Tropical Diseases (CTD), Sacro Cuore Hospital, Negrar, Verona, Italy; 2 Coordinating Resources to assess and improve health status of migrants from Latin America (COHEMI) project study group, European Commission, Health Cooperation Work Programme, FP7 (GA-261495), Milan, Italy; 3 Department of Public Health, IRCCS—Mario Negri Institute for Pharmacological Research, Milan, Italy; 4 National Institute of Allergy and Infectious Diseases (NIAID), National Institutes of Health (NIH), Bethesda, Maryland, United States of America; 5 Instituto de Investigaciones en Enfermedades Tropicales—Universidad Nacional de Salta/CONICET, Oran, Argentina; 6 Barcelona Centre for International Health Research (CRESIB, Hospital Clinic-Universitat de Barcelona), Barcelona, Spain; Universidade Federal de Minas Gerais, BRAZIL

## Abstract

**Background:**

Traditional faecal-based methods have poor sensitivity for the detection of *S. stercoralis*, therefore are inadequate for post-treatment evaluation of infected patients who should be carefully monitored to exclude the persistence of the infection. In a previous study, we demonstrated high accuracy of five serology tests for the screening and diagnosis of strongyloidiasis. Aim of this study is to evaluate the performance of the same five tests for the follow up of patients infected with *S. stercoralis*.

**Methods:**

Retrospective study on anonymized, cryo-preserved samples available at the Centre for Tropical Diseases (Negrar, Verona, Italy). Samples were collected before and from 3 to 12 months after treatment. The samples were tested with two commercially-available ELISA tests (IVD, Bordier), two techniques based on a recombinant antigen (NIE-ELISA and NIE-LIPS) and one in-house IFAT. The results of each test were evaluated both in relation to the results of fecal examination and to those of a composite reference standard (classifying as positive a sample with positive stools and/or at least three positive serology tests). The associations between the independent variables age and time and the dependent variable value of serological test (for all five tests), were analyzed by linear mixed-effects regression model.

**Results:**

A high proportion of samples demonstrated for each test a seroreversion or a relevant decline (optical density/relative light units halved or decrease of at least two titers for IFAT) at follow up, results confirmed by the linear mixed effects model that showed a trend to seroreversion over time for all tests. In particular, IVD-ELISA (almost 90% samples demonstrated relevant decline) and IFAT (almost 87%) had the best performance. Considering only samples with a complete negativization, NIE-ELISA showed the best performance (72.5% seroreversion).

**Conclusions:**

Serology is useful for the follow up of patients infected with *S. stercoralis* and determining test of cure.

## Author Summary

Patients infected by *S*. *stercoralis* are at risk of fatal complications. It is therefore mandatory to demonstrate complete response to therapy. Post treatment evaluation should be done with highly sensitive diagnostic methods, which can exclude the persistence of the infection. Serology is more sensitive than fecal examination and coproculture. In this study, we compare the post-treatment performance of five serology tests, and suggest that they can be useful for the follow up of patients with *S*. *stercoralis* infection, especially in non-endemic areas, where there is no risk of reinfection. In fact, the results of the tests show a progressive decrease, towards negativization, of the values (expressed in different units, depending on the specific test) through time.

## Introduction

*Strongyloides stercoralis* infection is widely distributed in tropical, subtropical countries and even in areas of temperate climate [1]. Strongyloidiasis probably affects at least 370 million people worldwide [2] and represents a threat for immunosuppressed people, who tend to develop the fatal complications of the infection [1,3]. Therefore, it is mandatory to diagnose the infection during the chronic phase, which is often indolent and can be more easily treated [3].

The diagnosis of *S*. *stercoralis* infection is characterized by poor sensitivity of fecal-based methods [4]. Therefore, other diagnostic tools have been developed and demonstrated better sensitivity [4,5]. Polymerase chain reaction (PCR) is still based on in-house techniques [6–8], performed only in reference centers, and is not necessarily more sensitive than fecal culture[9]. Serology is more sensitive, though not 100% specific [4]. Some serology kits are commercially available [10,11]. A high sensitivity is also necessary when evaluating the response to the treatment, as treatment failures leave the patient exposed to the risk of developing a potentially fatal, disseminated strongyloidiasis at any time in his/her life [2]. Negative fecal-based methods cannot safely exclude persistence of infection [4,12], therefore the follow up of infected patients should also rely on more sensitive techniques as markers of cure. Although some authors have observed a decline of optical density (OD)/titers of serology tests over time, a wider comparative evaluation has not been carried out so far, and a clear definition of cure has not yet been established [13–20]. We recently published the results of a study comparing the accuracy of five serologic tests for the diagnosis of *S*. *stercoralis* infection [5]: two commercial ELISA tests (Bordier ELISA, IVD-ELISA), two tests based on the recombinant antigen NIE (ELISA and luciferase immunoprecipitation system, LIPS) and one in-house indirect immunofluorescence antibody test (IFAT). The study demonstrated a good performance of the tests, and in particular NIE-LIPS demonstrated the best accuracy for the diagnosis of *S*. *stercoralis*. The same tests were also evaluated on sera collected pre and post treatment in the present study.

Thus, the aim of this study was to compare the performance of the five tests for the follow up of patients after treatment in order to identify if antibody decline could be used a surrogate marker for cure, in addition to stool negativization.

## Methods

### Study population and data collection

This was a retrospective study on archived, anonymized sera available at the Centre for Tropical Diseases (CTD). Samples were classified according to a composite reference standard (a procedure suggested for evaluation of diagnostic tests when there is no gold standard) [21,22] as a) positive: positive fecal tests and/or at least 3/5 positive serologic tests; b) negative: negative fecal tests and less than 3 positive results out of the 5 serologic tests.

The inclusion criteria were: samples resulting positive before treatment, according to the composite reference standard), and available follow up serum sample/s, from 3 to 12 months after treatment. Treatment administered was ivermectin (stat dose of 200 μg/kg), with the exception of 6 cases treated with thiabendazole (two daily doses of 25 mg/kg for two days) in the earlier period. The exclusion criterion was travel history to endemic areas between treatment and follow up. The results of stool examination/agar culture were registered and entered in the study database.

### Test methods

Parasitological tests used were: at least 3 stool samples examined with microscopy (formol-ether concentration) and Koga agar plate culture [23,24]. These methods were performed at the CTD. The serology tests evaluated were: the CTD in-house immunofluorescence technique (IFAT) [13], two commercial ELISA tests (Bordier ELISA [10] and IVD ELISA [11]) and two techniques based on the recombinant antigen NIE (NIE-ELISA [25] and NIE-LIPS [26]. IFAT and the two commercial ELISA tests were executed by senior staff of the CTD Negrar (Verona), Italy, while NIE-LIPS and NIE-ELISA were up to senior staff of the National Institute of Allergy and Infectious Diseases (NIAID) of the National Institutes of Health (NIH), Bethesda, US and of the Instituto de Investigaciones en Enfermedades Tropicales of the University of Salta/CONICET, Oran, Argentina. Lab staff were blinded to the patients’ data and to the results of the other tests.

### Definitions of response to therapy

Cure was operationally defined by negative composite reference standard (see above) at follow up or at least by: negative stool examination/coproculture and decrease of at least half of initial eosinophil count.

For the evaluation of each test, we assessed, over the denominator of patients cured according to the operational definition reported above: a) the proportion of initially positive tests that were negative at follow up; b) the proportion of those showing a decrease of at least half of initial OD/relative light units (RLU) values (for ELISA tests and LIPS, respectively) or decrease of at least two titers (for IFAT). This was taken as an empirical measure of response to therapy.

### Sampling

**Figure 1:**
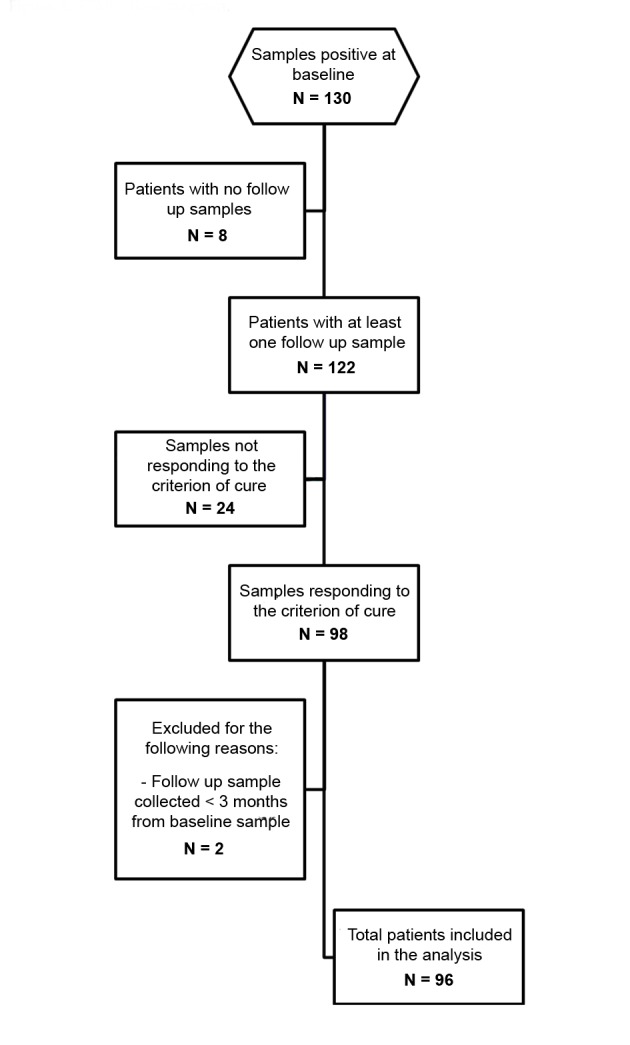
STARD flow chart, representing the selection of the study samples.

The STARD flow chart (Fig. 1) describes the selection of the samples tested. Among the 130 subjects responding to our definition of positive, 8 were excluded because follow up samples were not available. Of the remaining 122, 6 had a positive fecal result at follow up. Of the 116 testing negative at follow up, 98 met the criterion of cure as defined above, of which: 57 were negative according to the composite reference standard, and 41 showed a decrease of at least half of initial eosinophil count. Two subjects were excluded because their follow up sample was collected less than 3 months after the baseline sample. Eventually, 96 subjects were included in the analysis.

### Statistical methods

Primarily, the performance of each test was calculated as the proportion of samples demonstrating seroreversion or a quantitative decrease (as indicated above) over all positive samples (for the same test) at baseline. Uncertainty was quantified using 95% confidence intervals.

To reduce the limitations due to the different time intervals between treatment and observation (from 3 to 12 months), we used the following methods. The associations between the independent variables age and time and the dependent variable value of serological test (for all five tests), were analyzed by linear mixed-effects regression model. Linear mixed model is a generalization of traditional linear regression, which adjusts for the correlation between repeated measurements within each subject and finds the best linear fit to the data across all individuals [27,28]. More specifically, a unique identification number for each subject and time was treated as a random effect in the model and age was treated as fixed effect. Time was entered as random effect because measurements of the value of serological tests over time were not taken at regular time points. Interaction term between age and time was evaluated to include in the regression model by using Likelihood Ratio Test. Introduction of an interaction term is necessary where the effect of one variable (time) is affected by the presence or value of another variable (age). Unstructured covariance matrix was selected since this is the structure that appears to fit the data the best, based upon the Akaike’s information criterion (AIC).

Analyses were done by using SAS (version 9.1; SAS Institute, Inc, Cary, NC). We considered differences to be statistically significant when the p-value was <0.05.

### Ethical issues

Although this was a retrospective study on anonymously coded, cryo-preserved samples, the study protocol was nevertheless submitted to the Ethics Committee of the Coordinating Site (Comitato Etico Provinciale di Verona) for approval. The latter acknowledged the study protocol and formally authorized the study (protocol n. 13286/09.11.01 of 24^th^ April, 2012).

## Results

The sample selection and the laboratory analyses were performed during the second semester of 2012. The median age of the population considered was 42 years (IQ range 22.5–67). Table 1 shows the time (in months) elapsed from baseline to follow up. Every patient had a baseline evaluation both with serology and with parasitological methods. Only 9/96 (0.9%) patients had negative stools at baseline; according to the composite reference standard, these patients were included in the analysis because they had at least 3 out of 5 positive serologic results. All but these 9 patients, had also parasitological evaluation at the time of collection of the follow up serum sample. All had negative stool microscopy and culture at follow up (data not reported in Table 1), as this was the first required criterion for the definition of cure.

**Table 1:** Number of patients who had the follow up sample in each two-month period of time.

Months from baseline to follow up visit	Positive at fecal-based methods	Negative at fecal-based methods	Total
3–4	29	5	34
5–6	25	0	25
7–8	17	2	19
9–10	4	0	4
11–12	12	2	14
Total	87	9	96

For each time frame, it is also showed the number of patient who had positive versus negative stool microscopy and culture at baseline.

Table 2 shows, for each test, the percentage of serum samples showing response according to the pre-defined criteria. For each serologic test, we considered for this analysis only the samples that were positive at baseline. For instance, among the 96 samples resulting positive at baseline according to the composite reference standard, 91 had a positive IFAT result (see column “Positives at baseline”). The column “Negativization” comprises the samples which were positive at baseline and negative at follow-up, while the column “Response” includes the latter, plus the samples that, albeit remaining positive, showed a decrease of at least half of initial OD/relative light units (RLU) values (for ELISA tests and LIPS, respectively) or two titers (for IFAT). IVD-ELISA (almost 90% samples demonstrated response) and IFAT (almost 87%) had the best performance. When considering only samples with a complete negativization, NIE-ELISA showed the best performance (72.5% of seroreversion).

**Table 2:** Number of samples which demonstrated cure at follow up, for each test.

Test	Positives at baseline	Response	%	Negativization	%
IFAT	91	79	86.8	36	39.6
NIE-LIPS	82	65	79.3	35	42.7
NIE-ELISA	69	56	81.2	50	72.5
IVD	88	79	89.8	48	54.5
Bordier	86	71	82.6	47	54.7

Total baseline samples positive at composite reference standard: 96. Negativization concerns for each test the samples that were positive at baseline and negative at follow-up; response also includes samples that, albeit not yet negative at follow-up, showed a decrease in OD, RLU or titer, respectively, as explained in the text.

**Figure 2:**
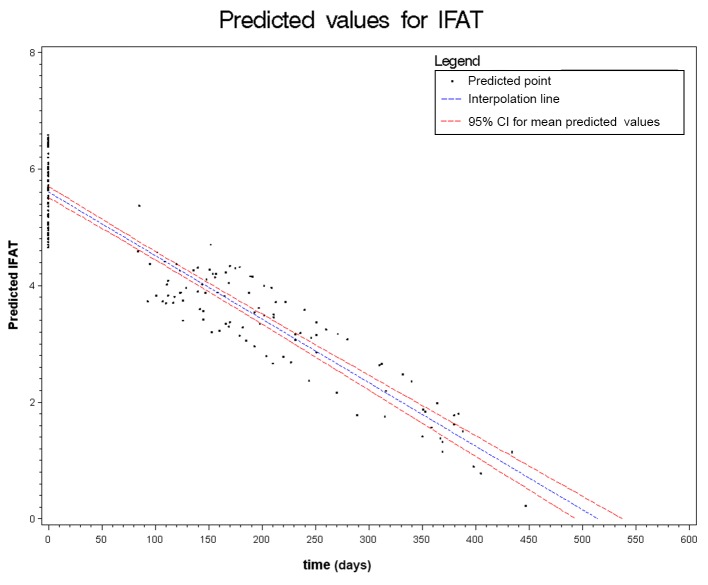
Results of the mixed effects model for IFAT.

**Figure 3:**
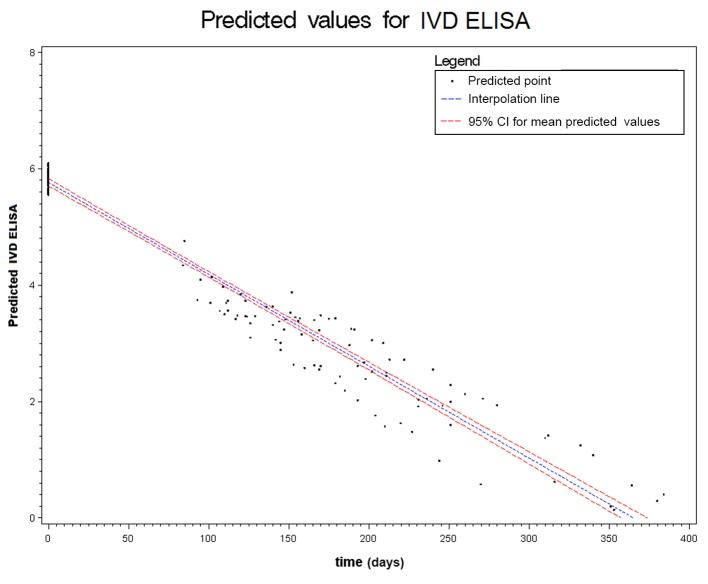
Results of the mixed effects model for IVD-ELISA.

**Figure 4:**
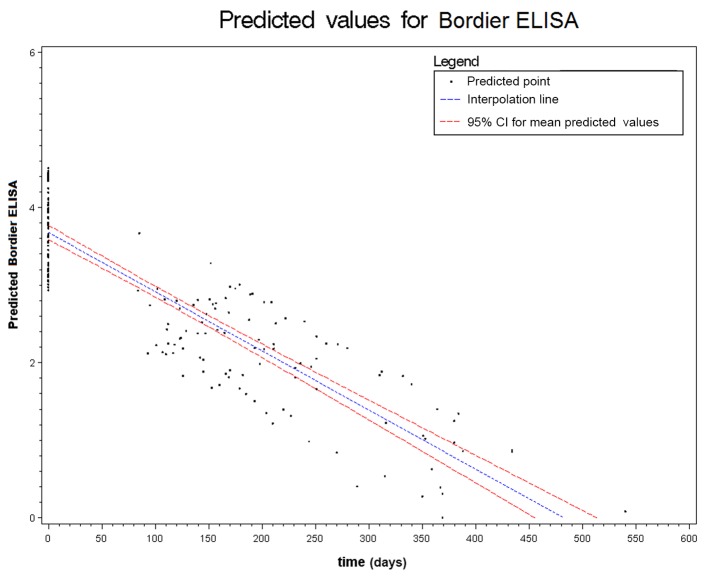
Results of the mixed effects model for Bordier-ELISA.

**Figure 5:**
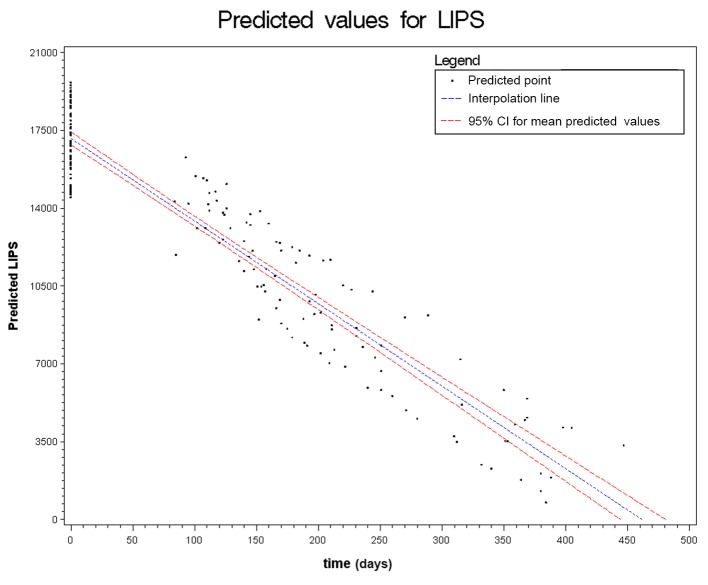
Results of the mixed effects model for NIE-LIPS.

**Figure 6:**
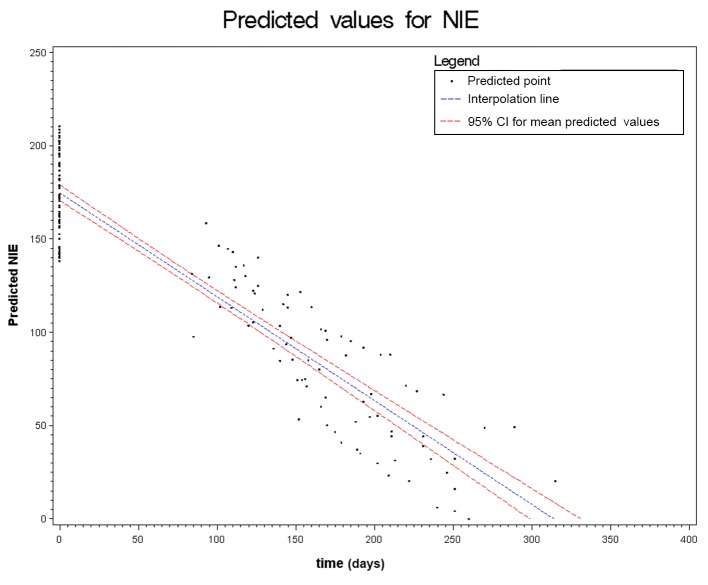
Results of the mixed effects model for NIE-ELISA.

Figs. 2 to 6 show the results of the mixed effects model for all five serological tests. They represent the prediction of the trends of the values of serology, from the baseline evaluation (0 on the x-axis) to the moment in which the result became negative (0 on the y-axis). Thus, significantly negative trends over time were detected for all tests. Moreover, the intersection of the interpolation line with the x-axis predicts the average time (days) required to obtain the negativization of the serology test. Therefore, NIE-ELISA and IVD-ELISA showed the most rapid predicted negativization (about 1 year from baseline evaluation).

Interaction terms between age and time were not statistically significant, meaning that effect of time was not affected by age in the outcome variable.

## Discussion

The results of this study indicate that serology tests for the diagnosis of strongyloidiasis tend to serorevert after effective treatment. All the tests evaluated demonstrated to be useful for monitoring, and the choice of a specific test is mainly influenced by diagnostic accuracy, costs and availability.

It is worth of note that the setting in which this study was performed excluded the possibility of re-infection, that is always possible in endemic areas. Therefore, the following recommendations are primarily applicable to non-endemic areas.

We suggest that serology, when affordable, should be routinely introduced in the diagnosis of strongyloidiasis, by virtue of its higher sensitivity, when compared with fecal methods. Serologic tests are the only available method to assess cure for patients with (false) negative fecal test results before treatment. Moreover, serology should be also performed in cases found positive in stool, in order to obtain a baseline result to be subsequently monitored at follow up. Negativization of fecal tests alone is not a sufficiently reliable marker of cure, due, again, to their sub-optimal sensitivity. It should also be considered that, while the excretion of larvae in stools stops within a few days after an effective treatment [29], it takes several months to demonstrate negativization of serology. Therefore, patients should be monitored at 6 and 12 months after treatment, to be able to demonstrate decrease and/or negativization of the serologic results, and thus be safely considered cured.

In areas where re-exposure can be excluded, a serological value failing to decrease should be cautiously interpreted as a treatment failure. In this case, the time-interval for evaluation after therapy is crucial, as our model shows that, especially for low values of OD/titer, it can be necessary to extend the follow up to more than 12 months. False positive results of serology might also be considered for those patients who do not show a response after one year, especially when the initial serology values were under a determined cutoff, as was showed by our previous study [5]. The possible cross-reactivity with other parasitic infections was also investigated in the same study and appeared to be of limited importance.

A combined diagnostic strategy (serology plus a suitable fecal method such as Baermann technique or Koga agar plate culture) is required at baseline evaluation, considering that a positive fecal result means 100% certainty of infection [4].

### Study limitations

Based on the operational case definition of cure, we obtained the denominator of “cured” patients on which we assessed the decline in titer of the different serologic tests. In the absence of a gold standard for cure, we cannot rule out that some patients might have been misclassified, i.e. considered cured when they were not, also considering that the eosinophil count can fluctuate. It is therefore possible that in some cases the lack of serologic response to cure could be due to misclassification. Moreover, the follow up samples were available at different time intervals after treatment, because of the retrospective design of the study. A three-month time could be a period of time too short to observe a decrease in the values of serology, therefore it cannot be excluded that a longer and more homogeneous period of observation would have demonstrated better performance of the tests in terms of percentage of seroreversion (as seen in Table 2). However, the application of the mixed effects model permitted to have a prediction of the decrease over time, making it possible to demonstrate a tendency to seroreversion for all tests. Another limitation is related to the different treatment used (ivermectin or thiabendazole). Although the two drugs demonstrated a comparable efficacy [30] we cannot exclude a difference in the rapidity of the response to treatment. However, the patients treated with thiabendazole were just a few (6 subjects), thus not allowing a separate analysis.

### Conclusion and further research needs

Our results demonstrate that each of the serology tests considered can be used for monitoring patients who received a treatment for *S*. *stercoralis* infection. Serology, in combination with fecal-based methods, should be used as the preferred tool for the follow up. Validation of PCR techniques for the follow up might be a useful support for situations of uncertainty (such as patients with serology values that do not seem to decrease over time). Further investigations are necessary to extend these considerations to endemic areas, where re-infection might be an issue.

## Supporting Information

S1 Supporting Information Comprehensive database. (MDB)
